# Interplay between Electric Field Strength and Number of Short-Duration Pulses for Efficient Gene Electrotransfer

**DOI:** 10.3390/ph17070825

**Published:** 2024-06-23

**Authors:** Ernestas Urbanskas, Baltramiejus Jakštys, Justinas Venckus, Paulina Malakauskaitė, Ingrida Šatkauskienė, Inga Morkvėnaitė-Vilkončienė, Saulius Šatkauskas

**Affiliations:** 1 Research Institute of Natural and Technological Sciences, Vytautas Magnus University, 44404 Kaunas, Lithuania; ernestas.urbanskas@vdu.lt (E.U.); baltramiejus.jakstys@vdu.lt (B.J.); justinas.venckus@vdu.lt (J.V.); ingrida.satkauskiene@vdu.lt (I.Š.); 2 Faculty of Electronics, Vilnius Gediminas Technical University, 10105 Vilnius, Lithuania; paulina.malakauskaite@imcentras.lt; 3 Department of Immunology and Bioelectrochemistry, State Research Institute Centre for Innovative Medicine, 08406 Vilnius, Lithuania; 4 Department of Nanotechnology, State Research Institute Centre for Physical Sciences and Technology, 02300 Vilnius, Lithuania; inga.vilkonciene@ftmc.lt

**Keywords:** electroporation, gene electrotransfer, transfection efficiency, cell viability, pulse intensity, pulse number

## Abstract

Electroporation is a method that shows great promise as a non-viral approach for delivering genes by using high-voltage electric pulses to introduce DNA into cells to induce transient gene expression. This research aimed to evaluate the interplay between electric pulse intensity and 100 µs-duration pulse numbers as an outcome of gene electrotransfer efficacy and cell viability. Our results indicated a close relationship between pulse number and electric field strength regarding gene electrotransfer efficacy; higher electric pulse intensity resulted in fewer pulses needed to achieve the same gene electrotransfer efficacy. Subsequently, an increase in pulse number had a more negative impact on overall gene electrotransfer by significantly reducing cell viability. Based on our data, the best pulse parameters to transfect CHO cells with the pMax-GFP plasmid were using 5 HV square wave pulses of 1000 V/cm and 2 HV of 1600 V/cm, correspondingly resulting in 55 and 71% of transfected cells and maintaining 79 and 54% proliferating cells. This shows ESOPE-like 100 µs-duration pulse protocols can be used simultaneously to deliver cytotoxic drugs as well as immune response regulating genetically encoded cytokines.

## 1. Introduction

Various methodologies, such as viral, chemical, and physical, are employed for DNA transfer into cells and tissues. One of the best-known physical DNA delivery techniques is electroporation (EP), which uses high-voltage (HV) electric pulses to introduce DNA into cells to induce transient, or permanent, gene expression if applied together with the CRISPR/Cas9 system and is called gene electrotransfer (GET) [1,2,3,4]. GET represents a promising approach for tackling diseases by editing specific target genes, thereby playing a crucial role in precision medicine, vaccination, genome editing, and boosting antitumor properties [5,6]. A significant challenge in ensuring the efficacy of gene therapy is the effective delivery of therapeutic genes into targeted cells and tissues [7]. Different protocols have been designed for gene electrotransfer in both in vitro and in vivo applications [8,9,10]. GET can be applied to most cell types and can be an alternate technique to other gene transfer methods but requires specialized equipment and optimization of procedure protocols [11].

Electroporation creates an electric potential difference between two electrodes, generating a uniform electric field with strength E determined by the voltage and distance between the electrodes [12]. Square-wave pulses are most used with parallel plate electrodes for GET. Transfection efficiency is mainly controlled by pulse parameters, such as electric field strength, pulse duration, delay between pulses, and the number of pulses [13,14]. Efficient transfer of small molecules into mammalian cells is achieved through the application of electric field strengths ranging from 100 to 1000 V/cm, pulses spanning from microseconds to milliseconds, and up to 10 repeating pulses at approximately 1 Hz frequency [12,15]. Three different combinations of pulse parameters are used to transfer larger molecules: (1) employing pulse amplitudes of up to a few kV/cm, with durations ranging from a few µs to several hundred microseconds [16,17]; (2) using low amplitude pulses of a few hundred V/cm, but with durations extending into the tens of milliseconds [18]; and (3) employing a combination of brief high-intensity pulses and long duration low-intensity pulses [15,19,20].

Studies have attempted to improve the efficiency of gene electrotransfer by modifying electric field settings, pulsing buffers, plasmid concentrations, and the shape and material of the electrodes [21,22,23]. However, optimization of the electric field parameters and plasmid concentration has been found to be the most effective for improving gene electrotransfer efficiency [24,25]. Studies have demonstrated that factors such as the duration of the square-wave pulse, the quantity of pulses, the strength of the field, and the frequency of pulse application can collectively impact gene delivery through electroporation [26,27,28].

Although GET is optimized mostly for using longer (1–20 ms) duration pulses [18,29,30], only a few studies aimed at optimizing GET using short-duration (~100 µs) pulses. These types of pulses are of interest also because they resemble the ESOPE protocol established for antitumor electrochemotherapy [31,32,33]. Therefore, to boost immune response after electrochemotherapy, ESOPE-like pulse protocols can be used simultaneously to deliver cytotoxic drugs as well as immune response regulating genetically encoded cytokines. Consequently, with this study, we aimed to determine the interrelation between different electric field strengths and 100 µs-duration pulse numbers regarding GET efficiency and cell viability. In this work, we emphasize an optimal number and voltage of the 100 µs duration square HV pulses for an efficient pMax-GFP electrotransfer into Chinese hamster ovary (CHO) cells.

## 2. Results

**Figure 1:**
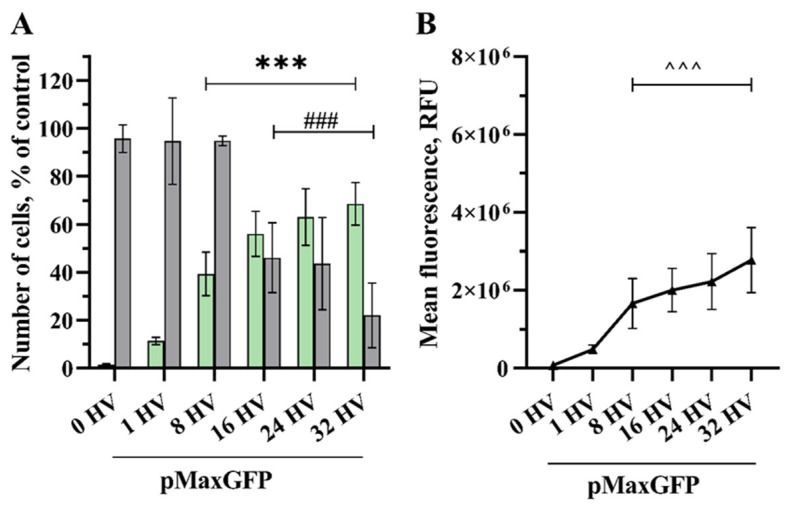
The percentage of cells transfected (GET) (**A**–green bars), the viability of the CHO cells (**A**–grey bars) and MFL (**B**) dependency on the number of electrical pulses. The electric field strength 800 V/cm, 100 µs duration, and a repetition frequency of 1 Hz. Bars and symbols represent average ± STDEV Bonferroni-corrected, where ***, ###, ^^^ denote *p* < 0.001, *n* = 6.

Increasing the number of 800 V/cm high voltage (HV) pulses resulted in a rising number of transfected cells and their fluorescence intensity (Figure 1). Here, one HV pulse of 800 V/cm was insufficient to obtain a significant increase in transfection efficiency compared to the control.

A significant increase in transfected cells (39%) compared to the control was achieved after exposure using 8800 V/cm HV pulses, where cell viability remained at 95% and MFL 1.7 × 10^6^ RFU (related fluorescence units). Although the highest transfection efficiency was achieved using 32 HV pulses, reaching 68%, the cell viability here dropped to 22%. When exposed to 16 HV pulses, transfection efficiency and MFL were 56% and 2.0 × 10^6^ RFU, respectively, maintaining cell viability at 46%.

However, transfection efficiency significantly differed when comparing the quantity of transfected cells in the control group with the cells treated with 8 to 32 HV pulses, the same as MFL, yet there was no significant rise in GET between 16 and 32 HV pulses. No noticeable decrease in cell viability was observed when using up to 8 HV pulses; however, using a higher number of pulses reduced cell viability drastically, the same viability was observed using 16 and 24 HV pulses, with an even higher decrease in cell viability when using 32 HV.

**Figure 2:**
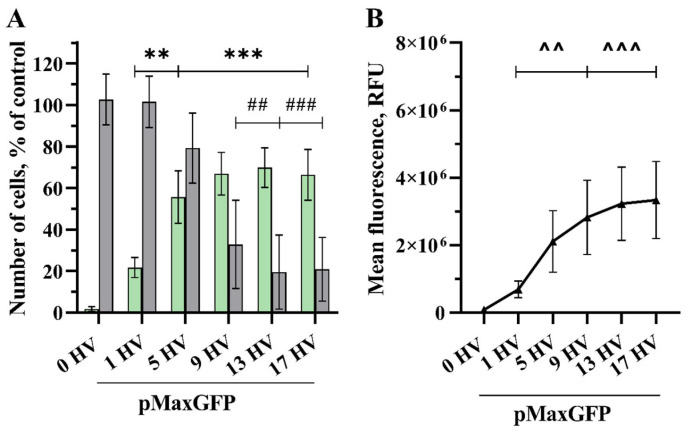
The percentage of cells transfected (GET) (**A**—green bars), the viability of the CHO cells (**A**—grey bars) and MFL (**B**) dependency on the number of electrical pulses. The electric field strength 1000 V/cm, 100 µs duration, and a repetition frequency of 1 Hz. Bars and symbols represent average ± STDEV Bonferroni-corrected, where ***, ###, ^^^ denote *p* < 0.001 and **, ##, ^^ denote *p* < 0.01, *n* = 6.

One HV pulse of 1000 V/cm was sufficient to significantly increase the number of transfected cells (22% and 0.691 × 10^6^ RFU) compared to the control (Figure 2). Furthermore, transfected cell numbers steadily increased up to nine HV pulses, giving 67% and an MFL of 2.8 × 10^6^ RFU. A higher number of pulses had no significant effect. However, the highest transfection efficiency of 70% was achieved using 13 HV pulses with an MFL of 3.2 × 10^6^ RFU. When applying five HV pulses, the transfection efficiency of the cells was 56%, and the MFL reached 2.1 × 10^6^ RFU. Cell viability decreased slightly to 79% using 5 HV pulses, which shows the best balance between cell viability and GET, and then dropped drastically to 33% and 20% using 9 and 13 HV pulses, respectively.

**Figure 3:**
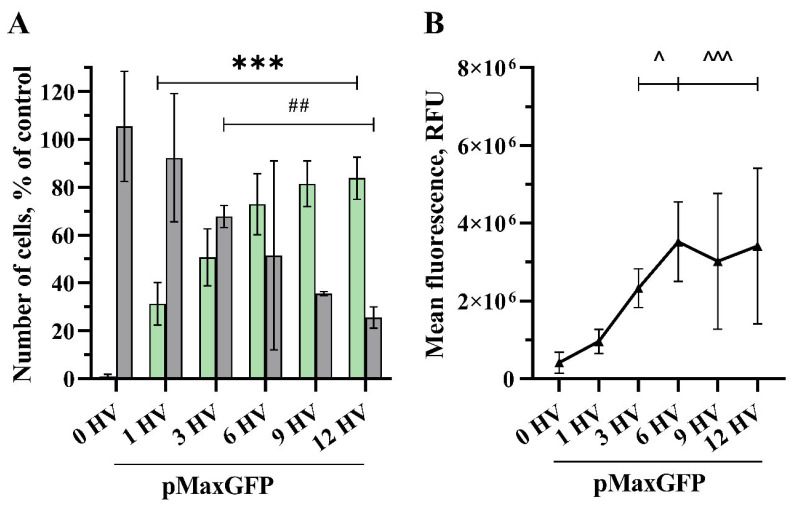
The percentage of cells transfected (GET) (**A**—green bars), the viability of the CHO cells (**A**—grey bars) and MFL (**B**) dependency on the number of electrical pulses. The electric field strength 1200 V/cm, 100 µs duration, and a repetition frequency of 1 Hz. Bars and symbols represent average ± STDEV Bonferroni-corrected, where ***, ^^^ denote *p* < 0.001; ## denote *p* < 0.01; and ^ denote *p* < 0.05, *n* = 6.

The quantity of transfected cells and the intensity of their fluorescence increased as the number of 1200 V/cm HV pulses increased (Figure 3). Applying 1 HV pulse of 1200 V/cm was sufficient to significantly increase the number of transfected cells (31% and 0.961 × 10^6^ RFU), while increasing the number of pulses resulted in higher transfection efficacy, peaking at 12 HV pulses with 84% transfected cell number and the MFL of 3.413 × 10^6^ RFU. No significant increase was observed comparing 6 and 12 HV pulses. Applying three HV pulses, transfection efficacy reached 51% with the MFL of 2.330 × 10^6^ RFU, but cell viability dropped significantly to 68%. The cell viability decreased with the increasing number of pulses applied, and 12 HV pulses resulted in a huge drop, with just 25% of viable cells.

**Figure 4:**
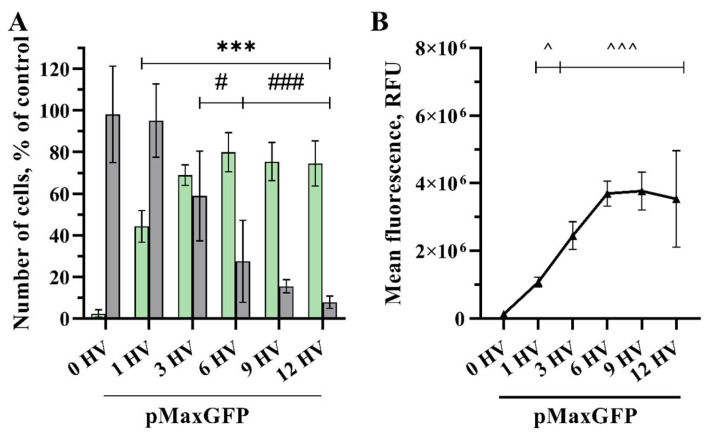
The percentage of cells transfected (GET) (**A**—green bars), the viability of the CHO cells (**A**—grey bars) and MFL (**B**) dependency on the number of electrical pulses. The electric field strength 1400 V/cm, 100 µs duration, and a repetition frequency of 1 Hz. Bars and symbols represent average ± STDEV Bonferroni-corrected, where ***, ###, ^^^ denote *p* < 0.001 and #, ^ denote *p* < 0.05, *n* = 6.

Transfection efficacy increased significantly when applying one HV 1400 V/cm pulse (44% and 1.076 × 10^6^ RFU) (Figure 4). Moreover, transfection efficacy increased up to six HV pulses and reached its highest percentage (80% with 3.693 × 10^6^ RFU). Applying three HV pulses, the transfection efficiency of the cells was 69%, and the MFL reached 2.5 × 10^6^ RFU. Increasing the number of pulses did not give any significant results compared with three HV pulses. Cell viability decreased to 59% using 3 HV pulses and then dropped drastically to 27%, 15%, and 8% using 6, 9, and 12 HV pulses, respectively.

**Figure 5:**
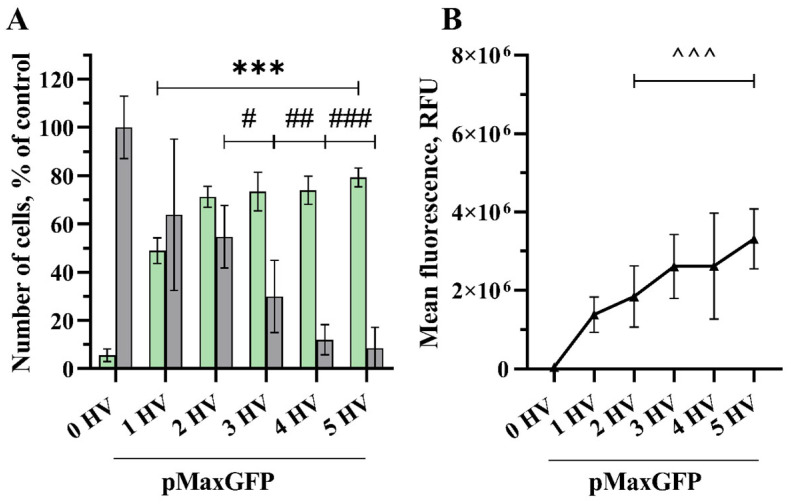
The percentage of cells transfected (GET) (**A**—green bars), the viability of the CHO cells (**A**—grey bars) and MFL (**B**) dependency on the number of electrical pulses. The electric field strength 1600 V/cm, 100 µs duration, and a repetition frequency of 1 Hz. Bars and symbols represent average ± STDEV Bonferroni-corrected, where ***, ###, ^^^ denote *p* < 0.001; ## denote *p* < 0.01; and # denote *p* < 0.05, *n* = 6.

Transfection efficiency was significant after applying a 1600 v/cm HV pulse (49% with 1.3 × 10^6^ RFU) and reached its highest when applying five HV pulses, reaching 79% with 3.3 × 10^6^ RFU (Figure 5). Although there was no significant difference observed in transfection efficacy between pulses from two HV to five HV pulses, the cell viability drop was noticeable even with one HV pulse but was not statistically significant. Applying more HV pulses drastically reduced cell viability, from 55% viable cells after the application of two HV pulses to only 9% after applying five HV pulses.

## 3. Discussion

Gene electrotransfer is a nonviral method for gene transfer into cells and tissues that exploits short high voltage electric pulses. The interest in this method’s applicability increased with time after proving GET efficacy, especially on nonadherent hard-to-transfect cells and tissues [10,12,15,25,34]. Most of the gene electrotransfer protocols utilize long pulses that are in the millisecond range (2–20 ms) [17,35]; differently, ESOPE protocol exploits short 100 µs pulses for electrochemotherapy to boost the uptake of chemotherapeutic medications [32,36]. Other EP applications also use short 100 µs pulses, such as irreversible electroporation [37,38], calcium electroporation [39,40], and gene electrotransfer combined with electrochemotherapy [41,42]. For instance, there are studies reporting gene electrotransfer in vivo comparing 100 V/cm 50 ms pulses vs. 900 V/cm 100 µs, indicating the superior long-lasting gene expression using 100 µs duration pulses [43]. However, there is a lack of comprehensive studies investigating short 100 µs pulses regarding gene electrotransfer while screening electrical field strength vs. pulse number for optimal parameters for GET efficiency and cell viability.

The primary intention of this research was to evaluate if excessive pulsing with a lower electric field strength can compensate gene electrotransfer compared to protocols using fewer pulse numbers of higher intensity when pulse duration is maintained. Previous studies typically used a range of 1 to 10 pulses of electric field strength varying from 100 to 1000 V/cm [10,12,44,45]. In our case, we first used lower-intensity pulses, repeating from 1 to 32 times. Secondly, we reduced the pulse number, followed by increasing the pulse intensity from 800 to 1600 V/cm.

**Figure 6:**
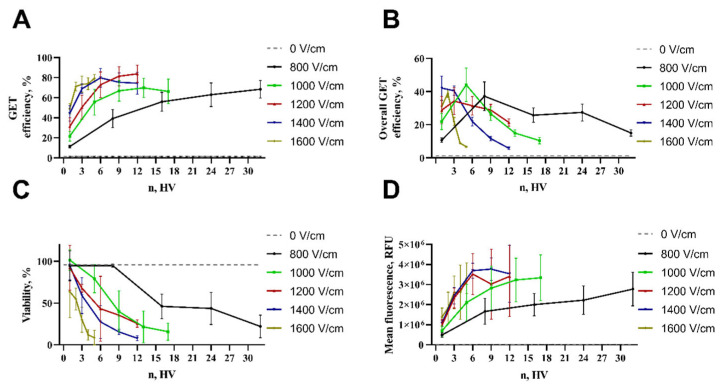
Summarized depiction of all results: transfection efficiency (**A**), overall GET (**B**), cell viability (**C**), and MFL (**D**) dependency on number of pulses and electrical field strength.

While several studies have demonstrated that effective DNA electrotransfer can be accomplished using short duration, high voltage (HV) pulses [16,43,46], other research suggests that longer pulses at intermediate voltages may yield more efficient DNA electrotransfer [18,30]. Haberl et al. investigated the efficiency of gene transfer using low voltage, long duration pulses (8 × 5 ms 0.7 kV/cm 1 Hz) compared to high voltage, short duration pulses (4 × 200 µs 1 kV/cm, 1 Hz), finding that long pulses provided better transfection efficiency with the highest yielding 60% of transfected cells with a viability of only 22% [3]. Similarly, Kandušer et al. conducted a study investigating the effect of high voltage (HV) and low voltage (LV) pulses on in vitro gene electrotransfer. They achieved the highest transfection efficiency using 1.4 kV/cm 4 × 200 µs 1 Hz, but it did not exceed 40% with a viability of 60% [10]. Furthermore, Potočnik et al. demonstrated that using high frequency bipolar pulses in vitro resulted in gene transfer levels reaching approximately 50% at most in both cases using 8 × 100 µs 1.6 kV/cm 1 Hz or 8 × 5 ms 0.5 kV/cm 1 Hz pulses with a preserved cell viability of 56% and 75%, respectively. In contrast, we could achieve transfection efficiencies as high as 82% using nine 1.2 kV/cm HV pulses and maintaining a cell viability of 36%. Moreover, we achieved higher transfection and similar viability with varying electric field strengths and number of pulses compared with the results obtained by other researchers. For instance, we obtained a GET efficiency of 71% while sustaining a cell viability of 55% using two 1.6 kV/cm HV pulses and 69% GET efficiency and 59% of viable cells employing three HV 1.4 kV/cm pulses (Figure 3, Figure 4 and Figure 5). To obtain a clear impression of transfection efficiency, overall GET, cell viability, and mean fluorescence intensity level (MFL) in dependence of pulse number and electrical field strength, the results are summarized in Figure 6.

Multiple reports show that higher electric field strength leads to better transfection but reduces cell viability [47,48,49]. Analogously to our research (Figure 6A), it was showed that when applying lower electrical field strength (800 V/cm), one pulse is not enough to give significant transfection efficiency [50], although a suitable number of pulses can lead to significant GET efficiency. According to our findings, a significant GET can be achieved with lower electrical field strength; the best transfection was achieved by applying a stronger electrical field and fewer pulses (Figure 6A). For instance, field strengths between 1.0 and 1.5 kV/cm are ideal for GET, but stronger electric fields result in increased cell death [16]. The primary cause of cell death following electroporation remains unspecified [51]. Nevertheless, the literature states several different mechanisms in regard to cell death after electroporation, such as irreversible membrane damage [52], ATP depletion [53], disruption of intercellular ion homeostasis [52], mitochondrial damage [54], increase of ROS [55], DNA damage [56], and protein damage [57] or loss of vital intracellular molecules [14].

It is known that a decrease in one pulse parameter can be compensated with an increase of another, resulting in the same transfection outcome. For instance, pulse intensity can be reduced by increasing pulse duration or pulse number [58]. Why higher electric field strength and fewer pulse numbers achieve a better balance between transfection efficiency and cell viability remains to be explained. We can speculate that although longer duration pulses can be more favorable to induce plasmid DNA movement, these pulses also lead to large pores [15] and, consequently, to larger molecules leaking out of the cells. Our results indicate that molecules like small nucleotides, proteins, and RNA can leak out following the electroporation. Thus, we can consider that longer duration pulses can induce higher pressure on loss of cell homeostasis and cell viability. In contrast, stronger electric field pulses of shorter duration largely affect the cell’s plasma membrane [15]; however, they result in the induction of smaller pores. Apparently, this can lead to less favorable interaction of plasmid DNA with the electroporated membrane; however, it is more appropriate for preserving cell viability. This can be of great practical interest for those who are looking for a high percentage of cell transfection with a lower cell viability or a lower percentage of cell transfection with a higher cell viability.

The reasoning behind choosing pulse parameters in our study was also based on ESOPE protocol parameters, where we compare ESOPE parameters with lower and higher pulse intensities. For this, we had to increase the pulse number with lower pulse intensities (800 V/cm) and reduce the pulse number with higher pulse intensities (1–1.6 kV/cm) to maintain viable cells. Moreover, we intended to screen a wide range of pulse numbers and intensities for their effect on GET efficiency and cell viability to provide a wider picture on the capability to apply these parameters in concert with ESOPE and GET for gene electrotransfer of immunomodulators [41].

All in all, pulse parameters for GET in regard to the electrical field strength and number of pulses should be selected according to the results one wants to obtain: high GET efficiency with reduced cell viability or low GET efficiency maintaining high cell viability.

## 4. Materials and Methods

### 4.1. Cell Culture

A Chinese hamster ovary cell line (CHO) obtained from the European Collection of Authenticated Cell Cultures (ECACC; 85050302) was utilized for the experiments. The cells were cultured in RPMI-1640 (Gibco, Gaithersburg, MD, USA ref. no. 21875) medium containing L-glutamine, supplemented with 1% Penicillin-Streptomycin (Gibco, Gaithersburg, MD, USA, ref. no. 15140-122) and 10% FBS (Gibco™, Gaithersburg, MD, USA, ref. no. A5256801) in a humidified atmosphere of 37 degrees Celsius and 5% CO_2_. Cells were passaged every 2–3 days and 24 h before each experiment and cultured in 10 cm petri dishes (TPP, Trasadingen, Switzerland, ref. no. 93100).

### 4.2. Plasmid DNA Preparation

To conduct the experiments, a pMAX-GFP (3.5 kb) (Lonza, Walkersville, MD, USA) that encoded the gene for green fluorescent protein was used. The plasmid was purified using the Plasmid Giga Kit (Qiagen, Hilden, Germany) in adherence to the manufacturer’s instructions. The purity (260/280 ratio 1.8 ± 0.1, 260/230 ratio 2.1 ± 0.1) and concentration of the nucleic acid were determined using a spectrophotometer (Nanodrop 2000, Thermo Fisher Scientific, Washington, DC, USA). DNA quality was evaluated using 1% agarose gel electrophoresis. To prevent plasmid degradation during storage, it was intended to prevent freezing-thawing cycles. In our case, pMAX-GFP was diluted in DNase free water after purification and diluted to final concentrations of 1 mg/mL and distributed into 1.5 mL centrifuge tubes (100 µL per tube).

### 4.3. Cell Harvesting and Electroporation

Before trypsinization, cells were washed once with 1× PBS (Sigma-Aldrich, St. Louis, MO, USA) and then treated with 2 mL Trypsin-EDTA solution (Sigma-Aldrich, St. Louis, MO, USA) at 37 degrees Celsius for 4 min. The cells were then resuspended in 2 mL of growth medium, centrifuged for 2 min at 300 RCF (LMC-3000) (Biosan, Riga, Latvia), and quantified using a hemocytometer (Paul Marienfeld GmbH & Co. KG, Lauda-Königshofen, Germany). Required number (2 × 10^6^) of cells were then resuspended in 1 mL electroporation buffer and washed twice. EP buffer specifications: 0.1 S/m, 270 mOsmol, 7.2 pH containing 1.68 mM MgCl_2_ (Sigma-Aldrich, St. Louis, MO, USA), 5.42 mM Na_2_HPO_4_ (Sigma-Aldrich, St. Louis, MO, USA), 2.91 mM NaH_2_PO_4_ (Sigma-Aldrich, St. Louis, MO, USA), and 270 mM sucrose (Sigma-Aldrich, St. Louis, MO, USA). Electroporation was carried out between two perpendicular stainless-steel plate electrodes separated by a 2 mm gap connected to the BTX T820 electroporator (Harvard Apparatus, Holliston, MA, USA) and a digital oscilloscope (Rigol DS2072A, Rigol Technologies Inc., Bedford, OH, USA). Oscilloscope was employed for the precise control of pulse parameters.

Brief GET procedure: a cell suspension of 54 μL containing 0.108 × 10^6^ cells was mixed with 6 μL of plasmid DNA solution (final concentration of 100 μg/mL) for each experimental point. Then, 55 μL of suspension was inserted between electrodes, and after pulsing, 50 μL (9 × 10^4^ cells) of suspension was transferred into a 24-well plate (TPP, Trasadingen, Switzerland) by tapping the electrodes into a dish bottom. Cells were then left to recover for 15 min. Afterwards, 450 μL of growing medium was added to each well. Then, 250 μL (4.5 × 10⁴ cells) of cell suspension was transferred into another 24-well plate prefilled with 250 μL of growing medium per well and left for 24 h to grow for GET evaluation. Subsequently, 44 μL (~8000 cells) of cell suspension was transferred from the primary plate into 1.5 mL tubes prefilled with 456 μL of growing medium. Then, 26 μL (~400 cells) of suspension was transferred into 40 mm Petri dishes (TPP, Transadingen Switzerland) for clonogenic assay for 6 days.

### 4.4. Assessment of GET Efficiency

Cells were washed with 1× PBS and suspended in 1× TrypLE Express Enzyme 1X (Thermo Fisher Scientific, Washington, DC, USA) 24 h post-electroporation and incubated for 10 min at 37 °C. Then, cells were analyzed using a BD C6 flow cytometer (BD Biosciences, Franklin Lakes, NJ, USA) to evaluate gene electrotransfer (GET) efficiency, mean fluorescence intensity level (MFL), and cell viability. GET was determined as the percentage of viable cells that were GFP-positive, while MFL was measured as the fraction of GFP-positive cells. The analysis was performed on 1 × 10^4^ cells for each experimental point at a flow rate of 66 µL/min using a 488 nm laser for excitation and an FL-1 emission filter (533/30 nm bandpass).

Normalization of transfection efficiency according to the number of alive cells was used to calculate the overall GET.

$$\mathrm{Overall}\mathrm{GET}=\frac{GET\%\times Viability\%}{100\%}$$

Cell Viability Using the Clonogenic Assay (CA): After 6 days, cells were fixed in 96% ethanol (Stumbras, Lithuania) for 10 min and stained with a crystal violet solution (Sigma-Aldrich, St. Louis, MO, USA). Using a light microscope (MBS-9 by LOMO, St. Petersburg, Russia), the number of cell colonies was determined and normalized to the control.

### 4.5. Statistical Analysis

Experiments were conducted at least three independent times as duplicates. The results obtained were presented as the mean value with the standard deviation. The statistical significance of differences between the groups was determined using an ordinary one-way ANOVA Bonferroni-corrected test.

## 5. Conclusions

Pulse intensity plays a higher role in increasing the transfected cell amount (GET efficiency) rather than pulse number (Figure 6A). Higher electrical field strength (1000–1600 V/cm) up to five pulses provides the optimal balance between the number of transfected cells and their viability (Figure 6B). GET efficiency increases with the increasing number of pulses (all tested electrical field strengths) with the expense of cell viability (Figure 6A,B); the overall GET efficiency, number of transfected cells, as well as mean fluorescence intensity level (amount of the pDNA transferred) using lower electric field strength can be compensated by the number of pulses (Figure 6). The highest transfection efficiency using a 100 µs duration pulse was obtained using five HV square wave pulses of 1000 V/cm and two HV of 1600 V/cm, correspondingly, resulting in 55 and 71% of transfected cells and maintaining 79 and 54% cell viability. This shows ESOPE-like pulse protocols can be used simultaneously to deliver cytotoxic drugs as well as immune response-regulating genetically encoded cytokines.

## Data Availability

Data are contained within the article.
